# Prehospital transport prioritization and time-varying selection bias in traffic-related multi-patient incidents: a nationwide retrospective cohort study

**DOI:** 10.1186/s13054-026-06196-2

**Published:** 2026-08-04

**Authors:** Atsushi Kubo, Shinobu Tamura, Atsushi Hiraide, Daigo Morioka, Ryu Murakami, Kenko Fukui, Shigeaki Inoue

**Affiliations:** 1 Faculty of Emergency Medical Science, Meiji University of Integrative Medicine, 2nd Floor, Building 6, Lecturer’s Office, 6-1 Hinoya, Honoda, Hiyoshi-cho, Nantan-shi, 629-0392 Kyoto Japan; 2 Department of Emergency and Critical Care Medicine, Wakayama Medical University, Wakayama, Japan; 3 First Department of Internal Medicine, Wakayama Medical University, Wakayama, Japan; 4 Department of General Medicine, Wakayama Rosai Hospital, Wakayama, Japan; 5 Faculty of Risk and Crisis Management, Chiba Institute of Science, Chiba, Japan

**Keywords:** Prehospital care, Triage, Transport prioritization, Selection bias, Causal inference, Mass casualty incidents, Trauma systems, Doubly robust estimation

## Abstract

**Background:**

In prehospital multi-patient traffic incidents, the first ambulance to depart —priority transport — may reflect underlying injury severity rather than a treatment effect, as more severely injured patients are systematically prioritized. Conventional statistical methods often fail to separate this severity-dependent selection from actual outcomes. We applied a “doubly robust” statistical framework (augmented inverse probability weighting, AIPW), which combines a treatment model and an outcome model, to better account for measured differences, reduce selection bias, and examine whether on-scene time modifies this association.

**Methods:**

We conducted a nationwide retrospective cohort study using emergency medical service transport records from the Fire and Disaster Management Agency of Japan (January 2021–December 2023). Of 13,821,224 records, 143,130 patients from traffic-related multi-patient incidents (TMIs; ≥2 patients per incident) with complete data were included. The exposure was priority transport. The primary outcome was physician-assessed death or critical/severe injury at hospital arrival. Marginal adjusted risk differences (RDs) were estimated using AIPW. Effect modification by on-scene time (5–60 min) was assessed by marginal standardization with cluster-bootstrap confidence intervals. Sensitivity analyses included overlap restriction and E-value quantification of unmeasured confounding.

**Results:**

Among 143,130 patients (median age 41 years; female, 50.57%), 62,769 (43.85%) underwent priority transport. Severe outcomes occurred in 4.35% of priority and 1.87% of non-priority patients. The overall RDs was 2.2% points (95% CI, 2.01–2.36). A significant interaction with on-scene time was observed (*p* < .001): the marginal RD was peaked at 5 min (6.4% points; 95% CI, 5.6–7.2), declined to a minimum of 1.4% points (95% CI, 1.3–1.6) at approximately 32 min, and increased modestly to 2.4% points (95% CI, 2.0–2.8) at 60 min. Sensitivity analyses confirmed result robustness, yielding an E-value of 3.76 (lower bound, 3.57).

**Conclusions:**

The association between priority transport and severe outcome was modified by on-scene time, with the largest risk difference at the shortest on-scene times. This U-shaped pattern is consistent with preferential selection of more severely injured patients for rapid transport, rather than a direct effect of transport order. Accounting for prehospital triage selection when interpreting transport-outcome associations in critical care research.

**Supplementary Information:**

The online version contains supplementary material available at https://doi.org/10.1186/s13054-026-06196-2.

## Introduction

The composition of patients arriving at critical care facilities from mass casualty events is determined, in part, by prehospital triage and transport prioritization decisions made under time pressure and resource constraints. Understanding how these decisions shape the severity distribution of patients reaching the intensive care unit is essential for interpreting outcome data and allocating critical care resources.

Traffic-related multi-patient incidents (TMIs) represent a high-stakes context in which this selection structure is most operationally visible, as competing patient needs require rapid prioritization under conditions of limited resources and time pressure [1, 2]. A central decision in this context is which of the patients to transport to hospital first. ‘Priority transport’ is defined here as the first patient departed from the scene with the intention of expediting definitive care for the most critically injured patient. However, there is limited evidence on whether this decision consistently improves outcomes and on how its association with outcomes varies with on-scene time. Investigative methods have been heterogeneous [3, 4].

Confounding by indication is a fundamental challenge in evaluating priority transport. Patients selected for first transport are systematically more severely injured than those transported later, and this underlying severity independently drives poorer outcomes. Established trauma scoring systems such as TRISS and the Revised Trauma Score incorporate physiologic parameters measured at hospital arrival, which are themselves downstream of prehospital triage decisions. Risk-adjusted outcome models based solely on hospital data may therefore incorporate prehospital selection artifacts, potentially misattributing differences in case-mix severity to hospital-level factors [5–7]. Importantly, however, this selection process is not static. As on-scene time increases, EMS personnel acquire more clinical information and operate under less acute time pressure, and this potentially may attenuates the severity differential between priority and non-priority patients [8]. Conventional analyses have not modeled this time-varying dimension of confounding, nor its implications for the interpretation of observed associations [9, 10].

Furthermore, TMIs present a distinct epidemiological setting from single-patient trauma. The presence of multiple patients competing for transport introduces structured selection processes that amplify confounding by indication beyond what is observed in routine EMS encounters [8]. This context motivates a study design that explicitly accounts for both the magnitude and the time-varying nature of this selection bias.

Whether priority transport—independent of injury severity—is associated with improved outcomes, and whether the time EMS providers spend on scene modifies this relationship, remains unresolved. To address this gap, we applied a doubly robust causal inference framework to estimate the population-average association between priority transport and severe clinical outcomes in traffic-related TMIs, and to examine heterogeneity in this association by on-scene time. This study aimed to: (1) estimate the association between priority transport and severe clinical outcome at hospital arrival in traffic-related multi-patient incidents, adjusting for measured covariates available in the national EMS registry; (2) examine whether on-scene time modifies this association; and (3) characterize the pattern of this association across the range of on-scene times as selection processes, including selection by injury severity.

## Methods

### Study design and data source

We conducted a retrospective cohort study using nationwide emergency medical service transport records administered by the Fire and Disaster Management Agency (FDMA) of Japan [11, 12]. These records are collected using a standardized Utstein-style data structure for systematic prehospital documentation across all prefectures. The Japan FDMA registry captures prehospital diagnostic codes, patient age, sex, time of call, incident size, and transport priority. However, it does not include granular patient-level clinical data, such as vital signs, Glasgow Coma Scale (GCS) scores, precise. These absences reflect the institutional structure of the administrative registry rather than an analytic choice. We analyzed data from between January 1, 2021 and December 31, 2023. The study was conducted in accordance with applicable guidelines for observational research; institutional review board approval was obtained and informed consent was waived owing to the observational design and deidentified data. We used the STROBE reporting guideline to draft this manuscript, and the STROBE reporting checklist when editing, included as Supplement 1 [13].

### Study population

Of 13,821,224 emergency medical service transport records captured nationwide during the study period, 1,048,494 traffic-related cases were initially identified. Traffic-related multi-patient incidents (TMIs) were defined as incidents involving two or more transported patients stemming from a single emergency call, which were identified by clustering individual transport records based on the unique fire department identifier, dispatch time, and specific incident location.

In the national Fire and Disaster Management Agency (FDMA) registry, these cases are uniformly classified under the broad administrative category of “traffic accidents,” which encompasses motor vehicle occupants, motorcyclists, cyclists, and pedestrians struck by motor vehicles; however, these specific sub-mechanisms cannot be granularly isolated or adjusted for as discrete independent variables due to registry coding structures.

**Fig. 1:**
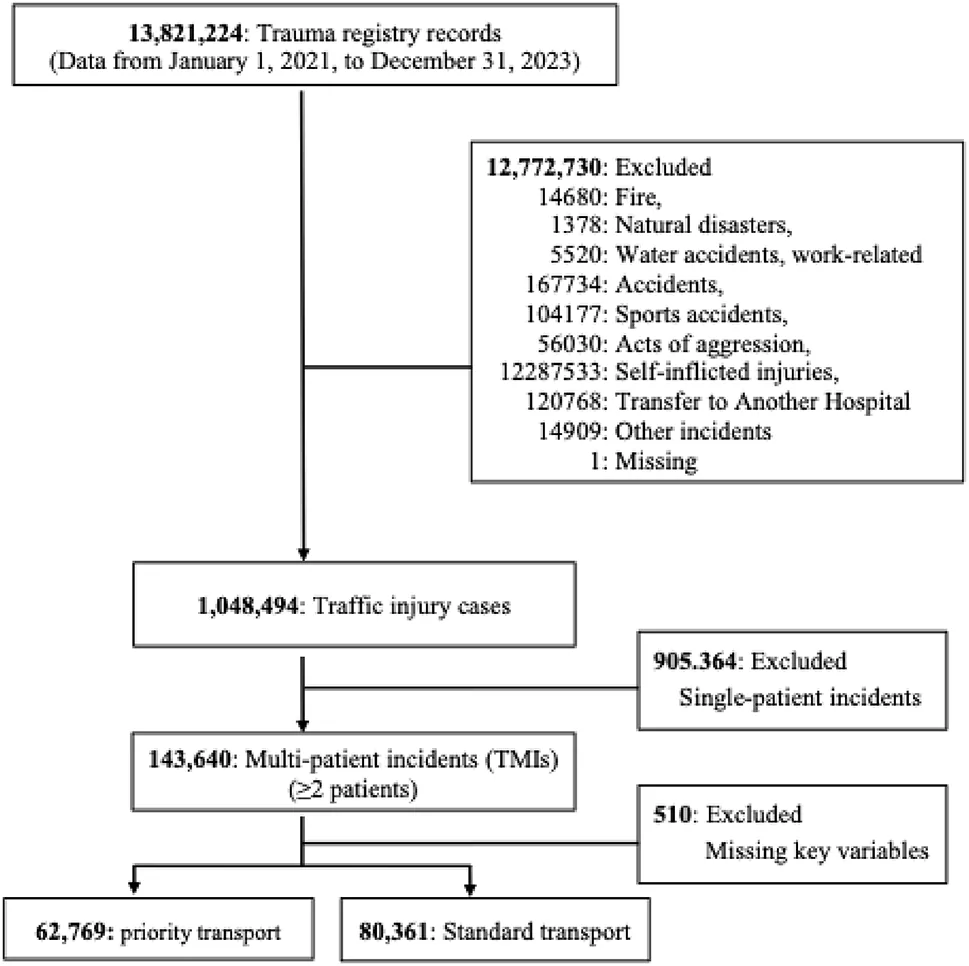
Study population flow diagram. Of 13,821,224 emergency medical service transport records, 12,772,730 were excluded (non-traffic incidents). This left 1,048,494 traffic injury cases, of which 905,364 were single-patient incidents and 143,640 formed traffic-related multi-patient incidents (TMIs; ≥2 patients per incident). After excluding 510 patients owing to missing key variables, 143,130 patients were included in the final analytic sample. Of these, 62,769 (43.85%) underwent priority transport (first patient transported from the scene) and 80,361 (56.15%) underwent standard transport

No lower age limit was applied, and pediatric patients (aged < 18 years; *n* = 28,308; 16.28%) were fully included in the primary analysis to reflect the comprehensive real-world case-mix of multi-patient field encounters. Regarding patients with absent vital signs or cardiac arrest, the Japanese EMS protocol dictates that individuals who are in cardiopulmonary arrest on EMS arrival, or who deteriorate into cardiac arrest during transport prior to hospital arrival, are tracked, recorded, and managed within a completely distinct, dedicated national out-of-hospital cardiac arrest (OHCA) registry utilizing the Utstein template. Because of this administrative sorting and data separation, patients with absent vital signs at or before hospital arrival were systematically excluded from the non-OHCA transport registry dataset utilized in this study, leaving an analytic sample comprised entirely of patients transported with signs of life. After the further exclusion of 510 patients with missing key variables regarding transport priority, on-scene time, or vital clinical outcomes, the final analytic dataset comprised 143,130 patients from traffic-related TMIs (Fig. 1).

Not all clustered incidents necessarily involved severe field resource constraints that would render formal, structured triage mandatory; the implications of this potential operational heterogeneity are addressed in the Limitations section.

### Exposure

The primary exposure was ‘priority transport’, the first patient transported from the scene within each incident. This operational definition serves as a pragmatic proxy for triage prioritization, but it may not fully capture clinical intent. Exposure misclassification is therefore possible, particularly in complex incidents where transport order is influenced by logistical constraints rather than clinical severity alone.

### Outcome

The primary outcome was severe clinical outcome, a physician-assessed severity rating at initial hospital evaluation using the registry classification scale (from 1 = death to 5 = mild). Severe outcome was defined as a severity score of 1 to 3 (death, critical, or severe injury, respectively). Death (score = 1), critical injury (score = 2), and severe injury (score = 3) were combined into a composite primary outcome because all three represent immediate life-threatening deterioration. The low frequency of isolated death (*n* = 455; 0.32% of the analytic cohort) precluded adequately powered analysis of mortality as a standalone endpoint. Outcomes for the individual severity categories are not presented separately because the primary objective of this study was to evaluate the composite severe outcome (death, critical injury, or severe injury). The low frequency of death alone (*n* = 455) limited statistical precision for component-specific analyses. The severity classification is derived from physician-assessed severity at initial hospital evaluation using the FDMA registry scale; this scale was not created by the investigators.

### Description of the Japanese EMS system

The Japanese emergency medical service (EMS) and trauma transport system operates within a centralized national framework but lacks a formally regionalized trauma system or a dedicated, exclusively designated trauma center network comparable to North American Level I or II trauma centers [14, 15]. Instead, definitive care for severe injuries is distributed across approximately 300 government-designated tertiary emergency medical centers nationwide, which manage all types of acute, life-threatening medical and surgical emergencies.

The prehospital system is governed by the Fire and Disaster Management Agency of the Ministry of Internal Affairs and Communications, with service delivery delegated to 750 municipal fire departments across Japan’s 47 prefectures, covering a total population of approximately 124 million over 378,000 square kilometers [14, 16]. The system is publicly financed, and emergency transportation is universally free of charge. Each ambulance is staffed by a three-person crew, including at least one Emergency Life-Saving Technician (ELST), a nationally licensed advanced prehospital provider. Approximately 50% of ELSTs possess additional authorization for advanced procedures, including endotracheal intubation and intravenous drug administration [17]. As of 2023, Japan maintains approximately 6,500 ambulances (roughly five per 100,000 population), with median nationwide EMS response times of approximately 10.3 min (urban areas: 7–9 min; rural areas: 10–15 min) [14, 18].

### Causal structure and covariates

A directed acyclic graph was constructed to clarify the assumed causal structure (eFigure 1 in the Supplement 1). Injury severity, the primary unmeasured confounder, was assumed to influence both priority transport assignment and severe outcome [19]. Pre-exposure covariates included age (natural cubic spline, 3 df), sex, time of call (sine and cosine of circular time to capture diurnal patterns), and incident size (2 patients vs. ≥3 patients). On-scene time was treated as an effect modifier rather than as a confounder or mediator. Although on-scene time may be influenced by EMS decision-making and incident complexity, conditioning on it in the propensity score model risks collider bias if it shares common causes with both priority transport and outcome. We therefore excluded on-scene time from the propensity score model and instead examined it as an effect modifier using marginal standardization across its range [20, 21].

In the FDMA registry, transport order and on-scene time are operationally linked because scene departure timestamps are recorded at the incident level. In supplementary analyses, 37,296 patients were identified from incidents in which two or more ambulances departed simultaneously, indicating that transport priority was not deterministically defined by departure timing alone. This operational structure further supported treating on-scene time as a contextual operational variable rather than a baseline confounder.

### Statistical analysis

#### Doubly robust estimation of marginal risk difference

Marginal adjusted risk differences were estimated using augmented inverse probability weighting (AIPW) to quantify the population-average association between priority transport and patient outcomes [22]. AIPW is a “doubly robust” method, meaning it provides valid estimates if either the propensity score model (predicting who gets priority transport) or the outcome model (predicting severe outcomes) is correctly specified [23, 24]. This approach offers stronger protection against model misspecification than standard logistic regression [22, 23, 25, 26].

The doubly robust AIPW estimator requires four core causal assumptions: (1) positivity, (2) consistency, (3) conditional exchangeability (that all confounders are measured and included), and (4) correct model specification for at least one component model. We explicitly acknowledge that conditional exchangeability is unlikely to be fully satisfied given the absence of physiologic injury severity data in the registry, meaning the estimated risk differences reflect covariate-adjusted associations rather than unbiased causal effects.

### Marginal RD curve by on-scene time

To evaluate how this relationship varies across operational contexts, we evaluated effect heterogeneity across on-scene time using marginal standardization [18]. Because the relationship between scene time and clinical outcomes may not be strictly linear, we modeled on-scene time flexibly using restricted cubic splines—a mathematical approach that allows the data to form smooth curves rather than forcing a straight line. We included an interaction term between transport priority and these scene-time splines in the outcome model.

Using this model, we calculated the predicted probability of a severe outcome for every patient in the dataset under two hypothetical scenarios: first, assuming everyone received priority transport, and second, assuming everyone received non-priority transport, fixing on-scene time at specific integer increments from 5 to 60 min. The difference between these two standardized probabilities defines the marginal risk difference (RD) at each time point. To avoid spline boundary instability, analyses were restricted to within 5–60 min; patients with an on-scene time < 5 min (*n* = 455) were excluded from the spline interaction analysis, while patients with an on-scene time > 60 min (*n* = 2,004) were right-censored at 60 min. To account for the fact that patients injured in the same incident share identical scene environments and logistical constraints, variance estimation and 95% confidence intervals (CIs) for the risk difference curve were computed using a cluster-based bootstrap technique at the incident level with 500 resamples. Overall population-level 95% CIs were generated using the standard sandwich estimator [27].

### Sensitivity and post-hoc analyses

To further ensure model robustness against potential unmeasured confounding and modeling assumptions, several pre-specified and post-hoc sensitivity analyses were performed. We repeated the primary analysis in an overlap-restricted population (propensity scores restricted to 0.1–0.9). Covariate balance was assessed using standardized mean differences (SMDs); SMD < 0.10 indicated adequate balance [23, 24, 28]. Additionally, we evaluated alternative specifications by adjusting for prehospital injury-severity proxy variables constructed from diagnostic codes, re-modeling incident size as a four-category ordinal variable (2, 3, 4, and ≥ 5 patients) to examine potential dose-response dynamics, and evaluating alternative functional forms for continuous covariates (including linear terms and alternative spline specifications for age and time of call). Finally, the E-value was calculated for the overall marginal RD to quantify the minimum strength of an unmeasured confounder required to fully explain away the observed association (detailed in eTable S1–S5 in the Supplement) [29].

### Statistical software

All analyses were performed using R version 4.5.2. The dplyr, splines, tidyr, car, and boot packages were used.

## Results

### Study population

After application of the eligibility criteria, 143,130 patients involved in traffic-related multi-patient incidents were included in the final analysis (Fig. 1). The median age was 41 years (IQR, 22–61 years), and 50.57% were female. Overall, 62,769 patients (43.85%) underwent priority transport. Baseline demographic and operational characteristics are presented in Table 1.

**Table 1:** Baseline characteristics of the study population

Characteristic	Overall (143,130)	Standard (80,361)	Priority (62,769)	SMD	Standard (weighted)	Priority (weighted)	SMD
Age, median (IQR)	41 (22–61)	36 (18–59)	45 (28–64)	0.307	42 (23–62)	42 (23–62)	0.021
Female sex, n (%)	72,385 (50.57)	41,064 (51.10)	31,321 (49.90)	0.024	50.30	50.35	0.001
On-scene time, median (IQR), min	22 (17–30)	23 (17–31)	21 (16–28)	N/A²	-	-	-
Incident size 2 patients, n (%)	99,984 (69.85)	49,985 (62.20)	49,989 (79.63)	0.389	71.69	72.66	0.010
Incident size ≥ 3 patients, n (%)	43,148 (30.15)	30,376 (37.80)	12,772 (20.35)	-	28.31	27.31	-

IQR, interquartile range; SMD, standardized mean difference

Weighted estimates were calculated using stabilized inverse probability weights. Effective sample size after weighting was 73,775 in the standard transport group and 57,144 in the priority transport group

On-scene time was considered a post-exposure operational variable and was therefore not included in the propensity score model; weighted balance statistics are not applicable

### Unadjusted clinical outcomes

The raw, unadjusted clinical outcomes according to transport priority are presented in Table 2. Severe clinical outcomes, defined as a physician-assessed severity category of death, critical injury, or severe injury at hospital arrival, occurred in 4,228 patients (2.95%) overall. The crude incidence of severe clinical outcomes was higher among patients who underwent priority transport than among those who received non-priority transport (4.35% [*n* = 2,729] vs. 1.87% [*n* = 1,499]).

**Table 2:** Unadjusted clinical outcomes according to transport priority

Outcome	Total (*N* = 143,130)	Priority (*n* = 62,769)	Non-Priority (*n* = 80,361)
Primary composite outcome, n (%)	4,228 (2.95)	2,730 (4.35)	1,499 (1.87)
Individual components			
Death alone, n (%)	455 (0.32)	299 (0.48)	156 (0.19)
Critical injury alone, n (%)	64 (0.04)	59 (0.09)	5 (0.01)
Severe injury alone, n (%)	3,709 (2.59)	2,363 (3.76)	1,338 (1.66)

Overall, entire study population with complete outcome data

The primary composite outcome was defined as a physician-assessed severity category of death, critical injury, or severe injury at hospital arrival. Severity categories were assigned by the attending physician at the receiving hospital according to the national emergency medical services reporting system

Among the individual components of the composite outcome, severe injury accounted for the majority of events (*n* = 3,709; 2.59% of all patients), followed by death (*n* = 455; 0.32%) and critical injury (*n* = 64; 0.04%). Crude rates of each outcome component were consistently higher in the priority transport group than in the non-priority transport group (Table 2).

Because transport priority is preferentially assigned to patients perceived to be at higher clinical risk, these unadjusted outcome comparisons should be interpreted as descriptive and not as estimates of the causal effect of priority transport.

### Covariate balance

Before weighting, priority and non-priority groups differed on age (median 45 vs. 36 years; SMD, 0.307) and incident size (2-patient incidents: 79.61% vs. 62.19%). After AIPW reweighting, all SMDs were < 0.10, indicating adequate balance. Propensity scores ranged from 0.068 to 0.790, with no evidence of positivity violations. The overlap-restricted population comprised 139,865 patients (97.72% of the analytic sample), confirming the rarity of extreme propensity scores.

### Overall marginal adjusted risk difference

The overall marginal adjusted RD was 2.2% points (95% CI, 2.01–2.36). In the overlap-restricted sensitivity analysis, the marginal RD was 2.0% points, suggesting that the result was not driven by patients with extreme propensity scores. The observed association is consistent with confounding by unmeasured injury severity, among other possible explanations, and should not be interpreted as a causal effect of priority transport (Table 3).

**Table 3:** Marginal causal effect estimates and heterogeneity by on-scene time

Measure	Estimate	95% CI
**Primary analysis (Crude severe outcome rate)**		
Severe outcome rate, priority, %	4.35	—
Severe outcome rate, non-priority, %	1.87	—
Marginal risk difference, %pts (overall)	2.20	2.01–2.36
**Sensitivity: overlap-restricted (PS 0.1–0.9)**		
N included No. (%)	139,865 (97.72)	—
Marginal RD, %pts	2.0	—
**Sensitivity: unmeasured confounding (E-value)**		
E-value (risk ratio)	3.76	—
E-value (lower CI bound)	3.57	—
**Sensitivity: TMLE with SuperLearner**		
Marginal RD, %pts	2.4	2.17–2.55
Relative risk	2.18	2.05–2.32
**Heterogeneity by on-scene time**		
Interaction (priority × on-scene time), p	< 0.001	—
Marginal RD at 5 min, %pts	6.4	5.6–7.2
Marginal RD at 10 min, %pts	3.8	3.5–4.1
Marginal RD at 20 min, %pts	1.8	1.6–1.9
Marginal RD at 32 min (minimum), %pts	1.4	1.3–1.6
Marginal RD at 40 min, %pts	1.5	1.4–1.7
Marginal RD at 60 min, %pts	2.4	2.0–2.8

AIPW, augmented inverse probability weighting; CI, confidence interval; PS, propensity score; RD, risk difference. Marginal RD at each on-scene time estimated by fixing scene time to the specified value for all patients and computing mean(mu1 − mu0) across the population; cluster-bootstrap 95% CIs (500 iterations at incident level). E-values calculated using the EValue R package (VanderWeele and Ding, 2017). TMLE, targeted minimum loss-based estimation; SuperLearner ensemble: generalized additive model and random forest (cv-SuperLearner)

### Heterogeneity by on-scene time

**Fig. 2:**
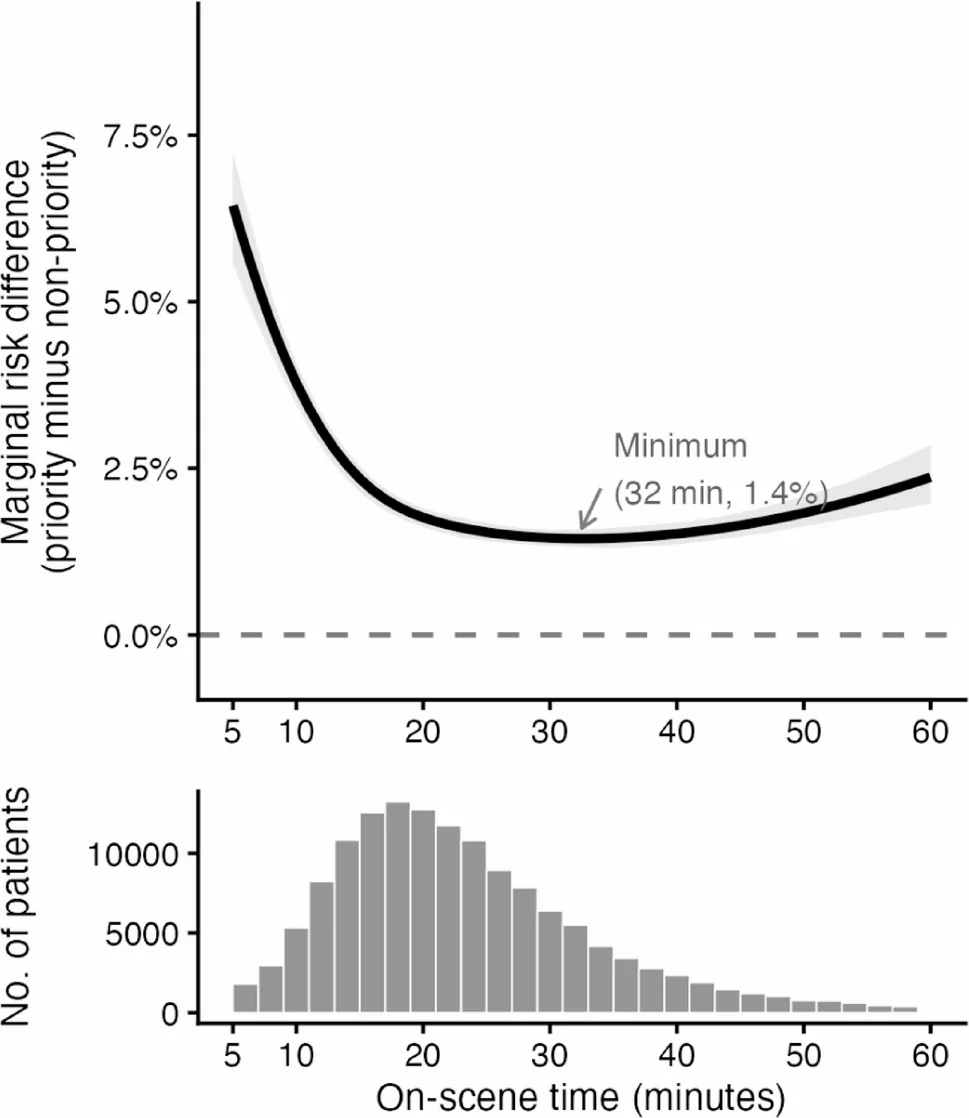
Marginal risk difference for severe clinical outcome by on-scene time and transport priority. The marginal risk difference (priority minus non-priority transport) is plotted against on-scene time (min). Marginal RDs were estimated using standardization across fixed levels of on-scene time. Shaded areas represent 95% confidence intervals estimated by cluster bootstrap resampling (500 iterations at the incident level). The marginal RD was 6.4 percentage points (95% CI, 5.6–7.2) at 5 min, declined to a minimum of 1.4 percentage points (95% CI, 1.3–1.6) at approximately 32 min, and increased to 2.4 percentage points (95% CI, 2.0–2.8) at 60 min. The risk difference remained positive throughout the observed range of on-scene times. This pattern is consistent with time-varying decision-making processes in which more severely injured patients are preferentially assigned to priority transport during shorter on-scene intervals. Because on-scene time is an operational variable that may itself be influenced by transport prioritization and incident management decisions, the curve should be interpreted as descriptive effect heterogeneity rather than a causal effect of modifying on-scene duration. The dashed horizontal line indicates a risk difference of zero. The bar chart at the bottom shows the distribution of on-scene time in the analytic sample

In an exploratory linear regression adjusted for injury-severity proxy variables, priority transport was associated with a shorter on-scene time (β = −5.09 min). A significant interaction between priority transport and on-scene time was observed (Wald test *p* < .001), indicating substantial heterogeneity in the marginal RD across the range of on-scene times examined (Fig. 2). The marginal RD was 6.4% points (95% CI, 5.6–7.2) at 5 min of on-scene time, declined progressively to a minimum of 1.4% points (95% CI, 1.3–1.6) at approximately 32 min, and increased modestly to 2.4% points (95% CI, 2.0–2.8) at 60 min. The marginal RD remained positive throughout the range of on-scene times examined, with no reversal of direction observed.

### Sensitivity analysis

The E-value for the overall marginal RD was 3.76 (lower CI bound, 3.57), indicating that an unmeasured confounder would need to be associated with both priority transport and severe outcome by a risk ratio of at least 3.57 to fully explain the observed association. The overlap-restricted marginal RD (2.0% points) was nearly identical to the primary estimate (2.2% points), indicating robustness to positivity violations. As a further sensitivity analysis, the targeted minimum loss-based estimation algorithm using cross-validated SuperLearner ensemble learners (generalized additive model and random forest) yielded a marginal risk difference of 2.4% points (95% CI, 2.17–2.55) and a risk ratio of 2.18 (95% CI, 2.05–2.32). Furthermore, sensitivity analyses adjusting for prehospital injury-severity proxy variables showed that the incorporating of these proxies led to only modest changes in the priority transport estimate (eTable S1 in the Supplement 1), and the full model coefficients for this severity-adjusted logistic regression are detailed in the Supplement (eTable S4 in the Supplement 1). Additionally, re-modeling incident size as a four-category ordinal variable (2, 3, 4, and ≥ 5 patients) yielded nearly identical estimates, demonstrating robustness to categorization assumptions (eTable S2 in the Supplement 1).

## Discussion

### Principal findings

In this nationwide cohort study of patients involved in TMIs, priority transport was associated with a modest increase in the probability of severe clinical outcomes at the population level. However, this association substantially varied across levels of on-scene time, exhibiting a U-shaped pattern. The magnitude of the association was greatest at short on-scene times (6.4% points at 5 min), attenuated at intermediate durations (1.4% points at 32 min), and increased modestly at longer durations (2.4% points at 60 min). Importantly, the direction of the association remained consistently positive across all time points examined.

### Interpretation: time-varying selection dynamics rather than treatment effects

The observed associations are consistent with confounding by unmeasured injury severity as one plausible explanation, among others, though a residual causal component cannot be excluded. Patients selected for early transport are likely to have more severe injuries than those transported later, and this severity differential may explain much of the observed excess risk [6, 7].

The U-shaped relationship between on-scene time and the priority transport–outcome risk difference does not imply that spending approximately 30 min on scene causally improves patient outcomes. Instead, we hypothesize that this pattern reflects a clinical and operational selection process where EMS providers rapidly transport the most critically injured patients first, causing a high intensity of selection bias at the shortest on-scene times. Specifically, at the shortest on-scene times, the selection process may be at its most intense—where triage decisions disproportionately prioritize the most critically injured patients for immediate departure—thereby maximizing the severity differential between priority and non-priority groups and producing the largest observed risk difference. While this prioritization simply reflects the triage system functioning appropriately as intended in clinical practice, it introduces a severe methodological concern for causal inference, as standard adjustment methods fail to achieve conditional exchangeability when such intense, severity-dependent selection operates dynamically. Conversely, patients who receive extended care on scene likely represent a less severely injured cohort, in whom providers feel clinically comfortable spending additional time to perform detailed assessments or elective interventions. This behavior naturally attenuates the severity differential between priority and non-priority groups, corresponding to the observed reduction in the marginal risk difference toward a minimum at approximately 32 min. The modest increase at the longest on-scene intervals may reflect a distinct subgroup of patients in whom extended duration is driven by complex extraction or extreme clinical instability rather than routine prehospital management. Therefore, this pattern represents severity-dependent selection into rapid transport, rather than a direct causal effect of on-scene time duration itself. Because we emphasize that because our dataset lacks direct empirical measures of baseline injury severity, these mechanistic explanations cannot be formally tested and must be interpreted with caution. This pattern is consistent with, but does not confirm, the hypothesis that injury severity differentially influences transport prioritization as on-scene time increases (Fig. 2) [30–32].

The marginal risk difference curve across on-scene time was generated by standardizing predicted outcomes at fixed levels of on-scene time across the population. Because on-scene time is a post-exposure operational variable that may itself be influenced by transport prioritization and incident characteristics, these estimates do not correspond to a well-defined intervention on on-scene time. Our objective was not to estimate the isolated causal effect of on-scene time. Rather, we sought to evaluate whether the association between transport prioritization and severe outcome differed across the range of observed on-scene times. Consequently, the interaction term should be interpreted as a descriptive assessment of effect heterogeneity across operational contexts rather than evidence of a mediating mechanism [33–36].

### Causal interpretation and identification limitations

Although a doubly robust estimation framework was applied, key assumptions required for causal identification are unlikely to be fully satisfied in this setting. In particular, physiologic injury severity—a major determinant of both transport prioritization and clinical outcome—was not directly measured. Residual confounding is therefore expected [37]. Accordingly, the primary estimand of this study should be understood as the covariate-adjusted association between transport prioritization and severe outcome rather than an unbiased causal effect of transport order itself. The use of causal inference methods in this study was intended to improve transparency regarding assumptions and to reduce measured confounding, while acknowledging that important unmeasured determinants of treatment assignment remain [35].

Sensitivity analyses partially addressed this concern. Adjustment for multiple injury-severity proxy variables produced only modest changes in the estimated marginal risk difference (eTable S1 in Supplement 1). Furthermore, incident-size sensitivity analyses using four categories (2, 3, 4, and ≥ 5 patients) yielded nearly identical estimates (2.07% vs. 2.09%; eTable S2 in Supplement 1). Functional-form sensitivity analyses for age and time-of-call specifications yielded qualitatively consistent positive associations despite moderate variation in effect size (eTable S3 in Supplement 1). Across all alternative specifications, the estimated marginal risk differences remained positive and within a similar range, supporting the robustness of the overall direction of the association while indicating some sensitivity of the effect magnitude to modeling assumptions [36].

As an additional sensitivity measure, the E-value was 3.76 (lower confidence bound, 3.57), indicating that an unmeasured confounder would need to be associated with both priority transport assignment and severe outcome by risk ratios of at least 3.57-fold each, beyond the measured covariates, to fully explain the observed association. However, whether physiologic injury severity meets or satisfies this threshold cannot be determined from the current dataset, as available registry data do not permit empirical quantification of this latent construct on the ratio scale. The E-value provides only a conceptual sensitivity framework and does not preclude a residual causal contribution; thus, these adjusted associations must be interpreted with strict caution regarding unmeasured confounding [30, 38].

### Interpretation of on-scene time standardization

The marginal risk difference curve across on-scene time was generated by standardizing predicted outcomes at fixed levels of on-scene time across the population. This approach does not correspond to a well-defined intervention on on-scene time, which is a post-exposure variable that is influenced by both clinical decision-making and operational factors. The resulting curve should therefore be interpreted as a descriptive tool that characterizes how the observed association varies across levels of on-scene time, rather than as an estimate of the causal effect of modifying on-scene duration [20, 22].

### Measurement considerations

The operational definition of ‘priority transport’ as the first patient transported from the scene provides a pragmatic proxy for triage prioritization but may not fully capture clinical intent. Misclassification of the exposure is therefore possible, particularly in complex incidents where transport order is influenced by logistical constraints rather than clinical severity alone. Such misclassification would likely bias estimates toward or away from the null in unpredictable ways [39, 40].

### Implications for research and practice

These findings point to a structural problem in prehospital observational research that extends beyond the specific context of transport prioritization. In emergency care settings, operational decisions are rarely made in isolation from patient severity: treatment assignment and the latent physiologic state driving outcomes are dynamically coupled throughout the prehospital encounter. This coupling means that observed associations between EMS interventions and outcomes may substantially reflect selection dynamics rather than treatment effects—even when doubly robust methods are applied and measured confounders are well balanced. Observational studies that do not explicitly model the time-varying structure of this selection process risk systematically misattributing dynamic bias to causal intervention effects [41].

This selection structure is most relevant to studies evaluating prehospital interventions (e.g., whole blood administration, prehospital airway management) or downstream hospital-based interventions in populations that have undergone prehospital triage, where severity-dependent selection operates dynamically. Conversely, studies examining diagnostic accuracy, triage algorithm development, or prognostic modeling may not require this bias to be explicitly modeled. By defining these boundaries, researchers can better distinguish between situations where selection dynamics threaten causal inference and those where operational prioritization reflects intended clinical practice without biasing the primary research question [42].

When detailed physiologic measurements or injury severity variables are unavailable, priority transport may serve as apragmatic surrogate marker of injury severity in observational EMS research. However, because its association with outcome varied according toon-scene time, future studies should consider modeling this relationship using interaction terms or flexible functional forms when detailedtemporal information is available. In settings where granular physiologic or injury severity data are available, priority transport should beregarded as complementary rather than a substitute for direct severity adjustment.

### Strengths and limitations

Strengths of this study include the use of a large nationwide registry, application of a doubly robust estimation framework, explicit assessment of covariate balance and overlap, and characterization of heterogeneity using marginal standardization with cluster-based bootstrap inference. Owing to the use of marginal rather than conditional estimates, the results reflect population-average associations [16].

Limitations of the study warrant careful consideration. First, residual confounding remains a key concern. Beyond physiologic injury severity, other unmeasured confounders likely include EMS crew experience and training level, hospital trauma capability at the receiving facility, geographic factors affecting transport time, and patient comorbidities; the AIPW estimator adjusts only for measured covariates. Second, potential exposure misclassification may arise from the operational definition of priority transport. Third, we applied an operational restriction of on-scene time to a 5–60 min range to avoid spline boundary instability (where values > 60 min were right-censored at 60 min). Furthermore, because the registry lacks granular timestamps for the exact moment of patient contact, on-scene time may partially include the brief interval between EMS arrival and initial assessment, potentially complicating its strict definition as a post-exposure variable. Additionally, the analysis of heterogeneity across on-scene times is descriptive in nature, and our mechanistic interpretation of the U-shaped pattern requires further validation in prospective or enriched data settings. Moreover, the registry includes physician-assessed severity at initial hospital evaluation but does not capturesubsequent in-hospital outcomes, such as hospital mortality or discharge disposition. Therefore, we were unable to evaluate whether prioritytransport was associated with downstream hospital outcomes. Finally, generalizability may be limited to EMS systems with similar operational structures to the Japanese system studied here [30].

Ultimately, interpreting registry-derived associations between prehospital interventions and outcomes demands rigorous attention to underlying selection structures. Future EMS registries that incorporate direct physiologic measurements—such as lactate, shock index, or field-based injury scoring—would substantially improve the validity of causal inference in this domain and the accuracy of severity adjustment at the point of critical care admission [42].

## Conclusions

In traffic-related multi-patient incidents, priority transport was associated with a higher probability of severe outcomes. This association varied substantially with on-scene time in a U-shaped pattern consistent with time-varying selection processes, potentially including confounding by injury severity, though a residual causal effect cannot be excluded. More broadly, these findings illustrate that in emergency care settings where treatment assignment is dynamically coupled with latent physiologic severity, observed associations between operational decisions and outcomes may substantially reflect selection dynamics rather than causal treatment effects—even under a doubly robust estimation framework. Therefore, explicit modeling of time-varying decision-making processes is essential when interpreting observational evidence in prehospital and acute care research, and prospective studies with direct measurement of physiologic severity remain necessary to enable definitive causal inference.

## Supplementary Information

Below is the link to the electronic supplementary material.

Supplementary Material 1: Figure S1, Tables S1-S5, STROBE Checklist.

## Data Availability

The data that support the findings of this study were provided by the Fire and Disaster Management Agency (FDMA) of Japan. Restrictions apply to the availability of these data, which were used under license for the current study. Data are available upon formal application and approval by the FDMA (contact via fdma-goiken@ml.soumu.go.jp).
