# Supplementary material for: Prehospital transport prioritization and time-varying selection bias in traffic-related multi-patient incidents: a nationwide retrospective cohort study
Source: Crit Care. 2026 Aug 4;30:524. doi: 10.1186/s13054-026-06196-2 (PMC13647583; doi:10.1186/s13054-026-06196-2)
Supplement: Supplementary file 1 [file 13054_2026_6196_MOESM1_ESM.docx]

**Supplementary Material 1**

**Prehospital Transport Prioritization and Time-Varying Selection Bias in Traffic-Related Multi-Patient Incidents: A Nationwide Retrospective Cohort Study**

Contents

eFigure S1. Directed acyclic graph illustrating the assumed causal structure. ………2
eTable S1. Comparison of models with and without injury-severity proxies	………3
eTable S2. Sensitivity analysis: incident size as four-category ordinal variable	………4
eTable S3. Sensitivity analysis: alternative functional forms for age and time of call	………5
eTable S4. Full model output: severity-adjusted logistic regression	………6

eTable S5. Prehospital Trauma Diagnostic Codes for Severity Proxy Construction　　　　　　.…...8-12
STROBE Checklist. Strengthening the Reporting of Observational Studies in Epidemiology. .….13-16

Among 174,024 patients included in the supplementary on-scene-time dataset, 30,894 (17.75%) originated from incidents in which two or more ambulances departed simultaneously. Because multiple ambulances departed at the same recorded time, transport order could not be uniquely determined from departure timestamps alone. These patients were therefore excluded from the primary transport-priority analysis but retained for descriptive evaluation of the operational relationship between transport priority and on-scene time. This finding indicates that transport priority was not solely defined by departure timing and likely reflected broader operational triage processes.

**eFigure S1. Directed acyclic graph illustrating the assumed causal structure**

Injury severity was assumed to influence transport prioritization, on-scene time, and severe outcome. Priority transport may subsequently influence on-scene time through operational decision-making. On-scene time was therefore treated as a post-exposure operational variable and evaluated as an effect modifier in outcome models rather than as a baseline confounder.

**eTable S1. Comparison of Models With and Without prehospital injury-pattern proxy variables**

Four binary injury-severity proxy variables (severe head injury, severe chest injury, severe abdominal injury, polytrauma) were derived from prehospital diagnostic codes and incorporated into both the propensity score model and outcome model. The table below compares model performance and the priority transport estimate before and after adjustment. Primary analysis result reported in the main manuscript was 2.20%.

Values shown here represent alternative model specifications used for sensitivity analyses.

| **Parameter** | **Base model (no severity proxies)** | **Severity-adjusted model** |
| --- | --- | --- |
| Marginal RD (95% CI) | 2.45 (2.34–2.55) | 2.32 (2.21–2.43) |
| Priority transport OR (95% CI) | 8.55 (5.04–15.1) | 4,68 (3.43–6.43) |
| Change in log-odds coefficient (%) | — | −0.96 |
| AIC | 345,263 | 323,973 |
| Likelihood ratio test p | — | < 0.001 |

OR = odds ratio; RD = risk difference; AIC = Akaike information criterion. The OR for priority transport reflects a local effect estimate at the spline reference value of on-scene time and is presented for model comparison purposes only; the marginal RD is the primary estimand. Full model coefficients for the severity-adjusted model are presented in eTable S4. Neither model corresponds to the primary AIPW analysis reported in the main manuscript. These analyses were performed solely to assess the influence of injury-severity proxy variables on effect estimates.

**eTable S2. Sensitivity Analysis: Incident Size as Four-Category Ordinal Variable**

The primary analysis categorized incident size as binary (2 patients vs. ≥3 patients). To assess robustness to this specification, incident size was re-modeled using four ordinal categories: 2, 3, 4, and ≥5 patients.

| **Model** | **Marginal RD (95% CI)** |
| --- | --- |
| Reference sensitivity model (binary: 2 vs ≥ 3 patients) | 2.07 (1.91–2.23) |
| Four-category sensitivity (2, 3, 4, ≥ 5 patients) | 2.09 (1.93–2.25) |

The estimated marginal risk difference was virtually unchanged (difference: 0.02 percentage points), indicating that the primary finding was not materially influenced by the binary categorization of incident size.

**eTable S3. Sensitivity Analysis: Alternative Functional Forms for Age and Time of Call**

The primary model specified age using a natural cubic spline with 3 degrees of freedom and time of call using sine/cosine terms. Sensitivity analyses examined alternative functional forms.

| **Specification** | **Marginal RD (95% CI)** |
| --- | --- |
| Reference specification (age spline 3 df; time sin/cos) | 2.32 (2.21–2.43) |
| Age linear; time sin/cos | 1.79 (1.63–1.95) |
| Age spline 2 df; time sin/cos | 1.86 (1.70–2.02) |
| Age spline 3 df; time quadratic | 1.83 (1.67–1.99) |

All alternative specifications produced positive marginal risk differences, although the magnitude of the estimates varied modestly across model specifications. These findings suggest that the direction of the association was robust to alternative functional-form assumptions for age and time of call, whereas the precise effect size showed some sensitivity to model specification. The reference specification shown here was estimated using the severity-adjusted sensitivity model including four injury-severity proxy variables. Therefore, estimates are not directly comparable to the primary analysis reported in the main manuscript.

**eTable S4. Full Model Output: Severity-Adjusted Logistic Regression**

The OR for priority transport (4.68) reflects a local effect estimate at the spline reference value of on-scene time, not an average effect. The marginal RD (2.32%, 95% CI 2.21–2.43%) is the primary estimand and is reported in the main text.

| **Covariate** | **OR** | **95% CI** | **p value** |
| --- | --- | --- | --- |
| Priority transport (at spline reference) | 4.68 | 3.43–6.43 | < 0.001 |
| Severe head injury | 8.47 | 7.12–10.04 | < 0.001 |
| Severe chest injury | 3.84 | 3.37–4.37 | < 0.001 |
| Severe abdominal injury | 32.4 | 27.61–38.0 | < 0.001 |
| Polytrauma | 17.36 | 15.02–20.04 | < 0.001 |

OR = odds ratio; CI = confidence interval. Values are from the severity-adjusted logistic regression model applied to the MTI analytic dataset (n = 143,130). The priority transport OR of 4.68 (95% CI 3.43–6.43) reflects a local conditional effect at the spline reference value of on-scene time and is not the primary estimand. The marginal RD (2.32%, 95% CI 2.21–2.43%) is the primary estimand and is reported in the main text. Severity proxy ORs reflect independent associations with the outcome adjusted for all other model covariates.

**eTable S5. Prehospital Trauma Diagnostic Codes for Severity Proxy Construction**

| **Code** | **Injury Type** |
| --- | --- |
| 21 | Amputation / Avulsion (Category) |
| 2101 | Avulsion - Ear / Nose |
| 2102 | Amputation - Hand / Finger |
| 2103 | Amputation - Wrist |
| 2104 | Amputation - Upper limb (excl. hand/finger/wrist) |
| 2105 | Amputation - Foot / Toe |
| 2106 | Amputation - Ankle |
| 2107 | Amputation - Lower limb (excl. foot/toe/ankle) |
| 2108 | Other Avulsion (Arterial rupture, external genitalia, etc.) |
| 22 | Contusion / Hematoma (Category) |
| 2201 | Contusion/Hematoma - Head |
| 2202 | Contusion/Hematoma - Face (Forehead, eye, cheek, nose, jaw, ear) |
| 2203 | Contusion/Hematoma - Neck (Throat, neck) |
| 2204 | Contusion/Hematoma - Chest |
| 2205 | Contusion/Hematoma - Abdomen |
| 2206 | Contusion/Hematoma - Back (incl. shoulder, scapula, lower back) |
| 2207 | Contusion/Hematoma - Buttocks |
| 2208 | Contusion/Hematoma - Genitalia |
| 2209 | Contusion/Hematoma - Upper limb |
| 2210 | Contusion/Hematoma - Lower limb |
| 23 | Open Fracture (Category) |
| 2301 | Open fracture - Cranium (Head) |
| 2302 | Open fracture - Facial bone |
| 2303 | Open fracture - Clavicle / Scapula |
| 2304 | Open fracture - Trunk (Spine: Cervical/Thoracic/Lumbar excl. L5) |
| 2305 | Open fracture - Pelvis (incl. L5, sacrum, coccyx, hip bone) |
| 2306 | Open fracture - Upper limb bone |
| 2307 | Open fracture - Lower limb bone |
| 2308 | Open fracture - Other sites |
| 24 | Closed Fracture (Category) |
| 2401 | Closed fracture - Cranium (Head) |
| 2402 | Closed fracture - Facial bone |
| 2403 | Closed fracture - Clavicle / Scapula |
| 2404 | Closed fracture - Trunk (Spine: Cervical/Thoracic/Lumbar excl. L5) |
| **Code** | **Injury Type** |
| 2405 | Closed fracture - Pelvis (incl. L5, sacrum, coccyx, hip bone) |
| 2406 | Closed fracture - Upper limb bone |
| 2407 | Closed fracture - Lower limb bone |
| 2408 | Closed fracture - Other sites |
| 25 | Dislocation / Sprain (Category) |
| 2501 | Dislocation/Sprain - Temporomandibular joint (Jaw) |
| 2502 | Dislocation/Sprain - Shoulder joint |
| 2503 | Dislocation/Sprain - Elbow joint |
| 2504 | Dislocation/Sprain - Wrist / Finger joint |
| 2505 | Dislocation/Sprain - Hip joint |
| 2506 | Dislocation/Sprain - Knee joint |
| 2507 | Dislocation/Sprain - Ankle / Toe joint |
| 2508 | Achilles tendon rupture |
| 2509 | Stiff neck, etc. (excl. cervical sprain) |
| 2510 | Other Dislocation/Sprain (Muscle strain, etc.) |
| 26 | Nerve / Spinal Cord / Brain Injury (Category) |
| 2601 | Brain injury (Traumatic brain injury) |
| 2602 | Thoracic spinal cord injury |
| 2603 | Lumbar spinal cord injury |
| 2604 | Nerve injury - Upper limb |
| 2605 | Nerve injury - Lower limb |
| 2606 | Injury such as cervical sprain (Whiplash) |
| 2607 | Injury such as thoracic sprain |
| 2608 | Injury such as lumbar sprain (Acute lumbago, herniated disc) |
| 2609 | Other nerve / spinal cord injuries |
| 27 | Open Wound (Category) |
| 2701 | Open wound - Head |
| 2702 | Open wound - Face (Eye, ear, eardrum, tongue, oral cavity, broken teeth, pharynx) |
| 2703 | Open wound - Neck, trachea, pharynx |
| 2704 | Open wound - Chest |
| 2705 | Open wound - Abdomen |
| 2706 | Open wound - Back (incl. shoulder, scapula, lower back) |
| 2707 | Open wound - Buttocks |
| 2708 | Open wound - Genitalia |
| 2709 | Open wound - Upper limb |
| 2710 | Open wound - Lower limb |
| **Code** | **Injury Type** |
| 2711 | Open wound - Other sites |
| 28 | Contused Wound / Laceration (Category) |
| 2801 | Laceration/Abrasion - Head |
| 2802 | Laceration/Abrasion - Face / Ear |
| 2803 | Laceration/Abrasion - Neck |
| 2804 | Laceration/Abrasion - Chest |
| 2805 | Laceration/Abrasion - Abdomen |
| 2806 | Laceration/Abrasion - Back (incl. shoulder, scapula, lower back) |
| 2807 | Laceration/Abrasion - Buttocks |
| 2808 | Laceration/Abrasion - Genitalia |
| 2809 | Laceration/Abrasion - Upper limb |
| 2810 | Laceration/Abrasion - Lower limb |
| 29 | Puncture Wound (Category) |
| 2901 | Puncture wound - Head |
| 2902 | Puncture wound - Face / Ear |
| 2903 | Puncture wound - Neck |
| 2904 | Puncture wound - Chest |
| 2905 | Puncture wound - Abdomen |
| 2906 | Puncture wound - Back |
| 2907 | Puncture wound - Buttocks |
| 2908 | Puncture wound - Genitalia |
| 2909 | Puncture wound - Upper limb |
| 2910 | Puncture wound - Lower limb |
| 30 | Gunshot Wound (Category) |
| 3001 | Gunshot wound - Head |
| 3002 | Gunshot wound - Face / Ear |
| 3003 | Gunshot wound - Neck |
| 3004 | Gunshot wound - Chest |
| 3005 | Gunshot wound - Abdomen |
| 3006 | Gunshot wound - Back |
| 3007 | Gunshot wound - Buttocks |
| 3008 | Gunshot wound - Genitalia |
| 3009 | Gunshot wound - Upper limb |
| 3010 | Gunshot wound - Lower limb |
| 31 | Incised Wound / Cut (Category) |
| 3101 | Incised wound/Cut - Head |
| **Code** | **Injury Type** |
| 3102 | Incised wound/Cut - Face / Ear |
| 3103 | Incised wound/Cut - Neck |
| 3104 | Incised wound/Cut - Chest |
| 3105 | Incised wound/Cut - Abdomen |
| 3106 | Incised wound/Cut - Back |
| 3107 | Incised wound/Cut - Buttocks |
| 3108 | Incised wound/Cut - Genitalia |
| 3109 | Incised wound/Cut - Upper limb |
| 3110 | Incised wound/Cut - Lower limb |
| 32 | Chop Wound (Category) |
| 3201 | Chop wound - Head |
| 3202 | Chop wound - Face / Ear |
| 3203 | Chop wound - Neck |
| 3204 | Chop wound - Chest |
| 3205 | Chop wound - Abdomen |
| 3206 | Chop wound - Back |
| 3207 | Chop wound - Buttocks |
| 3208 | Chop wound - Genitalia |
| 3209 | Chop wound - Upper limb |
| 3210 | Chop wound - Lower limb |
| 33 | Internal Injury / Organ Damage (Category) |
| 3301 | Intracranial injury (Concussion, brain contusion, intracranial hemorrhage, brain edema) |
| 3302 | Pneumothorax / Hemothorax |
| 3303 | Myocardial / Pulmonary contusion (excl. rupture) |
| 3304 | Intra-abdominal organ injury (Stomach, bowel, others; excl. rupture) |
| 3305 | Pelvic organ injury (excl. rupture) |
| 3306 | Cardiac rupture / Pulmonary rupture |
| 3307 | Intra-abdominal organ rupture (Stomach, bowel, others) |
| 3308 | Pelvic organ rupture |
| 34 | Foreign Body / Accidental Ingestion (Category) |
| 3401 | Foreign body in eye |
| 3402 | Foreign body in ear |
| 3403 | Foreign body in nose |
| 3404 | Foreign body in pharynx / larynx |
| 3405 | Foreign body in bronchus / lung |
| 3406 | Foreign body in oral cavity |
| **Code** | **Injury Type** |
| 3407 | Foreign body in esophagus / stomach |
| 3408 | Foreign body in genitourinary tract |
| 35 | Drowning / Submersion |
| 36 | Burns / Scalds (Category) |
| 3601 | Severe burns (By area, face/extremities, airway burn, or with soft tissue/bone damage) |
| 3602 | Moderate burns (2nd degree: 15-30%, or 3rd degree: ≤10%) |
| 3603 | Minor burns (2nd degree: ≤15%, or 3rd degree: ≤2%) |
| 37 | Asphyxiation / Suffocation (Category) |
| 3701 | Asphyxia - Anoxia / Oxygen deficiency |
| 3702 | Asphyxia - Airway obstruction / occlusion (by external pressure) |
| 38 | Poisoning (Category) |
| 3801 | Drug poisoning / Overdose |
| 3802 | Food poisoning (Mushroom, oyster, pufferfish, etc.; excl. bacterial) |
| 3803 | Gas poisoning / Carbon monoxide |
| 3804 | Other poisoning |
| 39 | Multiple Trauma / Polytrauma (Category) |
| 3901 | Multiple trauma - Whole-body contusion and fracture |
| 3902 | Multiple trauma - Head and trunk fractures |
| 3903 | Multiple trauma - Head and upper limb fractures |
| 3904 | Multiple trauma - Head and lower limb fractures |
| 3905 | Multiple trauma - Open wound + Fracture |
| 40 | Others (incl. non-medical facilities) (Category) |
| 4001 | Others - High/Low atmospheric pressure (Altitude/Caisson/Decompression sickness) |
| 4002 | Others - Motion sickness |
| 4003 | Others - Starvation / Dehydration, or diagnosed as no abnormality |
| 4004 | Others - Animal/Insect bites and stings (Centipede, snake, spider, bee, etc.) |
| 4005 | Others - Sequelae (incl. postoperative sequelae) |
| 4006 | Others - Miscellaneous |
| 41 | Unknown Diagnosis, etc. |

The table lists the prehospital trauma diagnostic codes used to construct the severity proxy in the severity-adjusted sensitivity analysis. These codes were assigned by emergency medical service (EMS) personnel during the completion of their activity reports, based on the definitive diagnoses confirmed after hospital arrival, and were grouped according to the primary anatomical injury region.

**STROBE Statement—checklist of items that should be included in reports of observational studies**

Manuscript: Prehospital Transport Prioritization and Time-Varying Selection Bias in Traffic-Related Multi-Patient Incidents: A Nationwide Retrospective Cohort Study
Study design: Retrospective cohort study | Journal: Critical Care | Date: 10 May 2026
https://www.strobe-statement.org/checklists/

|  | **Item No.** | **Recommendation** | **Page No.** | **Relevant text from manuscript** |
| --- | --- | --- | --- | --- |
| Title and abstract | 1a | *(a)* Indicate the study's design with a commonly used term in the title or the abstract | 1 | Title: "Prehospital Transport Prioritization and Time-Varying Selection Bias in Traffic-Related Multi-Patient Incidents: A Nationwide Retrospective Cohort Study." Abstract Design: "Retrospective cohort study (January 2021–December 2023)." |
|  | 1b | *(b)* Provide in the abstract an informative and balanced summary of what was done and what was found | 1 | Abstract provides study design, setting, participants, exposure, outcome, main results (marginal RD 2.2%pts, 95% CI 2.01–2.36; U-shaped heterogeneity by on-scene time), and conclusions noting confounding by injury severity. |
| Background/rationale | 2 | Explain the scientific background and rationale for the investigation being reported | 2 | Introduction paragraph 1–3 describes the operational challenge of transport prioritization in TMIs, the mechanism of confounding by indication, and the gap in understanding time-varying selection dynamics in this setting. |
| Objectives | 3 | State specific objectives, including any prespecified hypotheses | 2 | We applied a doubly robust causal inference framework to estimate the marginal population-level association between priority transport and severe clinical outcomes in traffic-related TMIs, and to characterize how this association varies with on-scene time. Study objective was to estimate the marginal association between priority transport and severe outcome and to assess heterogeneity by on-scene time. |
| Study design | 4 | Present key elements of study design early in the paper | 2–3 | Methods section begins: We conducted a retrospective cohort study using a Nationwide emergency medical service transport records administered by the Fire and Disaster Management Agency of Japan, collected using a standardized Utstein-style data structure (FDMA)." Design, setting, and time period stated in first paragraph of Methods. |
| Setting | 5 | Describe the setting, locations, and relevant dates, including periods of recruitment, exposure, follow-up, and data collection | 3 | “Nationwide emergency medical service transport records administered by the Fire and Disaster Management Agency (FDMA) of Japan. These records are collected using a standardized Utstein-style data structure for systematic prehospital documentation across all prefectures. Data from January 1, 2021, through December 31, 2023.” Japanese EMS System section describes the governance, staffing, and operational characteristics. |
| Participants | 6a | *(a) Cohort study—*Give the eligibility criteria, and the sources and methods of selection of participants. Describe methods of follow-up | 3 | Study Population section: “Of 13,821,224 emergency medical service transport records, 1,048,494 traffic-related cases were identified. TMIs were defined as incidents involving ≥2 patients and were identified by clustering records based on fire department identifier, dispatch time, and incident location. This yielded 143,130 patients from traffic-related TMIs after excluding 510 patients with missing key variables (Figure 1).” |
|  | 6b | *(b) Cohort study—*For matched studies, give matching criteria and number of exposed and unexposed | N/A | Not applicable. This is not a matched study. Comparison is between priority (n=62,769) and non-priority (n=80,361) transport within the same incident-based registry cohort. |
|  | **Item No.** | **Recommendation** | **Page No.** | **Relevant text from manuscript** |
| Variables | 7 | Clearly define all outcomes, exposures, predictors, potential confounders, and effect modifiers. Give diagnostic criteria, if applicable | 3 | Exposure: "Priority transport, defined as being the first patient transported from the scene within each incident." Outcome: "Physician-assessed severity rating at initial hospital evaluation (1=death, 2=critical, 3=severe, 4=moderate, 5=mild); severe outcome defined as score 1–3." Covariates: age (ns, 3df), sex, time of call (sin/cos), incident size. DAG describes assumed causal structure (eFigure 1). On-scene time: effect modifier. |
| Data sources/measurement | 8* | For each variable of interest, give sources of data and details of methods of assessment. Describe comparability of assessment methods if there is more than one group | 3 | All variables derived from FDMA nationwide emergency medical service transport records. Physician-assessed severity recorded at hospital arrival. Scene time computed from dispatch and departure timestamps. Incident key derived by clustering on fire department ID, dispatch time, and location. Comparability of assessment is ensured by the standardized national data structure across all fire departments. |
| Bias | 9 | Describe any efforts to address potential sources of bias | 3–4 | Causal Structure and Covariates section explicitly addresses confounding by indication via DAG construction and AIPW. Propensity score estimated with pre-exposure covariates only (on-scene time excluded as post-exposure variable). IPW trimmed at 99th percentile. Overlap restriction sensitivity analysis (PS 0.1–0.9) performed. E-value calculated for unmeasured confounding. Exposure misclassification acknowledged in Methods and Limitations. |
| Study size | 10 | Explain how the study size was arrived at | 3 | This was a registry-based study using all available data meeting eligibility criteria over the 3-year study period (January 2021–December 2023). No formal sample size calculation was conducted; the analytic sample of 143,130 patients was determined by data availability after applying eligibility criteria. |
| Quantitative variables | 11 | Explain how quantitative variables were handled in the analyses. If applicable, describe which groupings were chosen and why | 3–4 | Age modeled with natural cubic spline (3 df) to allow non-linear associations. Time of call encoded as sine and cosine of circular time (2π × minutes/1440) to capture diurnal patterns without imposing linearity. On-scene time restricted to 5–60 minutes and modeled as natural cubic spline (4 df) in the outcome model interaction term. Incident size categorized as 2 patients vs ≥3 patients. |
| Statistical methods | 12a | *(a)* Describe all statistical methods, including those used to control for confounding | 4 | Augmented inverse probability weighting (AIPW): propensity scores from logistic regression (age ns3, sex, time sin/cos, incident size); stabilized weights trimmed at 99th percentile. Outcome model: logistic regression with counterfactual prediction. AIPW influence function applied: mean[μ₁−μ₀+A(Y−μ₁)/ps−(1−A)(Y−μ₀)/(1−ps)]. Population-average (marginal) estimate obtained. |
|  | 12b | *(b)* Describe any methods used to examine subgroups and interactions | 4 | Marginal RD curves were estimated across the observed range of on-scene time (5–60 minutes) by standardizing predicted outcomes under priority and non-priority transport for all patients at each time point. Effect heterogeneity was assessed using an interaction term between transport priority and on-scene time, and results were visualized in Figure 2. |
|  | 12c | *(c)* Explain how missing data were addressed | 3 | Patients with missing data on key variables (n=510) were excluded from the analytic sample. The nature of missing data is described in the Study Population section. Analysis was conducted on complete cases (N=143,130). |
|  |  |  |  |  |
|  | **Item No.** | **Recommendation** | **Page No.** | **Relevant text from manuscript** |
|  | 12c | *(c)* Explain how missing data were addressed | 3 | Patients with missing data on key variables (n=510) were excluded from the analytic sample. The nature of missing data is described in the Study Population section. Analysis was conducted on complete cases (N=143,130). |
|  | 12d | *(d) Cohort study—*If applicable, explain how loss to follow-up was addressed | N/A | Not applicable. This is a cross-sectional registry study of prehospital transport; follow-up ends at physician-assessed severity at hospital arrival. No longitudinal follow-up involved. |
|  | 12e | *(e)* Describe any sensitivity analyses | 4 | Three sensitivity analyses: (1) Overlap restriction (PS 0.1–0.9; n=139,865, 97.7% of sample); (2) TMLE with cross-validated SuperLearner ensemble (GAM + random forest), yielding marginal RD 2.4%pts (95% CI 2.17–2.55); (3) E-value calculation for unmeasured confounding (E-value 3.76; lower CI bound, 3.57). |
| Participants | 13a | *(a)* Report numbers of individuals at each stage of study | 5 | Figure 1 flow diagram: 13,821,224 registry records → 1,048,494 traffic injury cases → 143,640 TMIs (≥2 patients) → 510 excluded (missing key variables) → 143,130 final analytic sample → 62,769 priority / 80,361 standard transport. |
|  | 13b | *(b)* Give reasons for non-participation at each stage | 5 | 12,772,730 records excluded due to non-traffic incidents (fire, natural disasters, water accidents, occupational accidents, sports accidents, self-inflicted injuries, other). 510 patients excluded for missing key variables. |
|  | 13c | *(c)* Consider use of a flow diagram | 5 | Figure 1 is a study population flow diagram showing all stages of inclusion and exclusion. |
| Descriptive data | 14a | *(a)* Give characteristics of study participants and information on exposures and potential confounders | 5 / Table 1 | Table 1: age median (IQR) [overall 41(22–61); priority 45(28–64); standard 36(18–59)], female sex [72,385 (50.6%)], on-scene time [priority 21(16–28); standard 23(17–31) min], incident size, severe outcome rate. SMDs before and after weighting reported. |
|  | 14b | *(b)* Indicate number of participants with missing data for each variable of interest | 3 | 510 patients excluded due to missing key variables. No further imputation performed. Complete-case analysis used for all primary and sensitivity analyses. |
|  | 14c | *(c)* Cohort study—Summarise follow-up time | N/A | Not applicable. Outcome assessed at a single time point (hospital arrival); no longitudinal follow-up. |
| Outcome data | 15* | Cohort study—Report numbers of outcome events or summary measures over time | 5 | Overall severe outcome rate 3.0% (4,228/143,130); priority group 4.3% (2,729/62,769); standard group 1.9% (1,499/80,361). Reported in Results Study Population section and Table 1. |
| Main results | 16a | *(a)* Give unadjusted estimates and, if applicable, confounder-adjusted estimates and their precision. Make clear which confounders were adjusted for and why | 5–6 / Table 2 | Unadjusted rates reported in Table 1 (priority 4.3% vs standard 1.9%). Marginal adjusted RD: 2.2%pts (95% CI 2.01–2.36) from AIPW. Adjusted for: age, sex, time of call, incident size (pre-exposure covariates justified via DAG). On-scene time excluded from PS model (post-exposure variable). Adjustment rationale described in Causal Structure and Covariates section. |
|  | 16b | *(b)* Report category boundaries when continuous variables were categorized | 4–5 | On-scene time was modeled continuously using a natural cubic spline (4 df) and evaluated across the observed range of 5–60 minutes. Results are presented graphically in Figure 2. |
|  | 16c | *(c)* If relevant, consider translating estimates of relative risk into absolute risk | 5–6 / Table 2 | Absolute marginal RD reported as primary measure (2.2 percentage points). Risk ratio (2.18, 95% CI 2.05–2.32 from TMLE) also reported. Marginal RD curve across on-scene time provides absolute risk estimates at clinically relevant time points (Table 2, Figure 2). |
|  |  |  |  |  |
|  |  |  |  |  |
|  | **Item No.** | **Recommendation** | **Page No.** | **Relevant text from manuscript** |
| Other analyses | 17 | Report other analyses done—eg analyses of subgroups and interactions, and sensitivity analyses | 5–6 / Table 2 | Heterogeneity by on-scene time was evaluated using standardized marginal RD curves across 5–60 minutes and an interaction Wald test (Figure 2). |
| Key results | 18 | Summarise key results with reference to study objectives | 7 | Discussion Principal Findings: "Priority transport was associated with a modest increase in the probability of severe clinical outcomes at the population level. However, this association varied substantially across levels of on-scene time, exhibiting a U-shaped pattern... Importantly, the direction of the association remained consistently positive across all time points examined." |
| Limitations | 19 | Discuss limitations of the study, taking into account sources of potential bias or imprecision. Discuss both direction and magnitude of any potential bias | 9 | Strengths and Limitations section: (1) Residual confounding from unmeasured physiologic injury severity—direction likely toward overestimation of excess risk; E-value 3.57 quantifies required confounding magnitude. (2) Exposure misclassification (operational definition may not capture clinical intent)—direction unpredictable. (3) For interaction analyses, on-scene time was trimmed at 60 minutes to reduce spline boundary instability. (4) Descriptive U-shaped heterogeneity requires further prospective validation. (5) Generalizability limited to EMS systems similar to Japan. |
| Interpretation | 20 | Give a cautious overall interpretation of results considering objectives, limitations, multiplicity of analyses, results from similar studies, and other relevant evidence | 7–9 | Causal Interpretation section explicitly states: "estimated risk differences should not be interpreted as unbiased causal effects of priority transport. Rather, they represent bias-affected population-level associations." Interpretation of On-Scene Time Standardization clarifies this is descriptive standardization, not a well-defined intervention. E-value framed as quantifying required confounding, not robustness of causal effect. |
| Generalisability | 21 | Discuss the generalisability (external validity) of the study results | 9 | "Generalizability may be limited to EMS systems with similar operational structures to the Japanese system studied here." Description of Japanese EMS System section provides context on system-specific features (FDMA governance, ELST staffing, universal free service, 6,500 ambulances, median response 8–9 min). |
| Funding | 22 | Give the source of funding and the role of the funders for the present study | 10 | "Funding/Support: None." No external funding. IRB: Meiji University of Integrative Medicine IRB (No. 2025-072). Data use authorized by FDMA of Japan. |

* Give information separately for exposed and unexposed groups in cohort studies and, if applicable, for cases and controls in case-control studies.
